# Is Parent–Child Disagreement on Child Anxiety Explained by Differences in Measurement Properties? An Examination of Measurement Invariance Across Informants and Time

**DOI:** 10.3389/fpsyg.2018.01295

**Published:** 2018-07-31

**Authors:** Thomas M. Olino, Megan Finsaas, Lea R. Dougherty, Daniel N. Klein

**Affiliations:** ^1^Department of Psychology, Temple University, Philadelphia, PA, United States; ^2^Department of Psychology, Stony Brook University, Stony Brook, NY, United States; ^3^Department of Psychology, University of Maryland College Park, College Park, MD, United States

**Keywords:** measurement invariance, anxiety, development, parent–child agreement, assessment

## Abstract

There are numerous empirical studies demonstrating that agreement between parent-reports of youth and youth self-reports of internalizing behavior problems is modest at best. This has spurred much research on factors that influence the magnitude of associations between informants, including individual difference characteristics of the informants and contexts through which individuals interact with the child. There is also tremendous interest in understanding symptom trajectories longitudinally. However, each of these lines of work are predicated on the assumptions that the psychometric construct that is being assessed from each informant and at each measurement occasion is the same. This study examined measurement invariance between maternal and child reports and longitudinally across ages 9 and 12 on five dimensions of anxiety using the Screen for Child Anxiety and Related Disorders (SCARED; Birmaher et al., 1999). No cross-informant models for anxiety dimensions achieved acceptable fit and at least partial metric and scalar invariance. Moreover, few longitudinal models demonstrated acceptable fit and at least partial metric and scalar invariance. Thus, using the SCARED as an example, these results show that inter-informant agreement may be compromised by different item functioning, and highlight the need for testing invariance before using measures for longitudinal tracking of symptoms.

## Introduction

There has been extensive research on agreement and disagreement between raters of symptoms of behavior problems in children and adolescents. These studies have examined multiple constellations of raters, including parents of the same target child, a parental caregiver and teachers, and parents and their child. Overall, there is modest agreement between parents and children and parents and teachers, but moderate agreement between parents (De Los Reyes et al., 2015). Attempts to understand factors that influence agreement between raters and also within raters over time have not provided complete explanations for lack of agreement. However, there have been no studies that test whether the underlying constructs reported by different informants, particularly primary caregivers and their children, are equivalent. There are few studies examining parallel issues over time. Without such evidence, it is difficult to interpret associations across informants as reflecting agreement on the same construct and how to evaluate longitudinal changes in the constructs. Thus, the present study examines whether measurement differences are present between parent- and child self-reports of anxiety that may partially explain lack of agreement across raters and across development.

The overall pattern of inter-informant agreement on child mental health symptoms have been extensively examined and summarized in two meta-analyses spanning a 28-year period. In the first, Achenbach et al. (1987) examined the associations between youth, parent, and teacher reports of internalizing and externalizing problems. In their work, there was stronger agreement among individuals with the same relationship to the target child (e.g., inter-parental agreement, average *r* = 0.61 across informant types), but more modest associations across different informant types (average *r* = 0.29 across all informants). Inter-informant agreement for overcontrolled and undercontrolled behavior problems, similar to internalizing and externalizing problems, respectively, were in the small-moderate range (*r*s = 0.32 and 0.41, respectively). More recently, De Los Reyes et al. (2015) conducted an updated analysis of studies since the Achenbach et al. (1987) paper. In this work, the authors found that the magnitude of interparental agreement (mean *r =* 0.59) was similar to that of other informant pairs with the same relationship to the target (i.e., teachers, mental health workers; average *r* = 0.58). However, agreement between raters with different relationships to the target was markedly lower (average *r* = 0.29). Overall inter-informant agreement was modest for both internalizing (*r* = 0.25) and externalizing problems (*r* = 0.30). The convergent findings from the two meta-analyses indicate that individuals with greater similarity in information will have a higher degree of similarity in their ratings of behavior. This has served as the foundation for the Operations Triad Model (De Los Reyes et al., 2013, 2015), which emphasizes context as an important factor in understanding reports of child behavior problems and assessing the incremental value of information from disparate sources.

Numerous studies have examined factors that explain the modest levels of convergence between informants on youth internalizing and externalizing behavior problems. These studies have considered moderating factors such as parent–child relationship functioning (Treutler and Epkins, 2003), parent symptoms (Youngstrom et al., 2000; Treutler and Epkins, 2003; Rothen et al., 2009), parental stress (Youngstrom et al., 2000; Langberg et al., 2010), child race (Youngstrom et al., 2000), child sex (Rothen et al., 2009), and characteristics of the symptoms themselves (e.g., observability, salience; Frank et al., 2000; Karver, 2006). However, these findings lack coherence and are sparsely replicated across samples.

There have been numerous studies examining the developmental course of anxiety disorders and symptoms with studies focusing on different age spans (Feng et al., 2008; Van Oort et al., 2009; Olino et al., 2010b, 2014). These studies have focused on risk factors predicting course as well as course predicting outcomes. However, there has been a paucity attention to longitudinal MI for youth anxiety. This precludes understanding whether observed mean-level changes are reflecting true score changes, or if these changes are influenced by changes in measurement properties. In one study (Mathyssek et al., 2013), the authors found evidence supporting MI for individual dimensions of anxiety from the Revised Child Anxiety and Depression Scale (RCADS; Chorpita et al., 2000). However, this study examined this issue using only youth-reports for a single assessment measure. Thus, comparisons between youth and parent reports across time are novel.

A key challenge in examining inter-informant agreement and assessing stability over time concerns the psychometric functioning of the measures used to assess the constructs. De Los Reyes et al. (2015) identified several sources of measurement error that may lead to attenuation of associations. Some of these are factors such as parental psychopathology or personality that may lead to distorted reports of youth behavior (Kagan, 1997; Najman et al., 2001; Hayden et al., 2010). Random error, such as imperfect test–retest reliability, could also limit the magnitude of associations across raters. Finally, the authors identify systematic error across informants as a potential explanation for the limited inter-informant associations.

Systematic error in ratings can come from several sources. De Los Reyes et al. (2015) focus on studies demonstrating differences in item response scaling as a possible, but unlikely, contributor to low inter-informant agreement. However, there are additional considerations that have not yet been explored in this area. For example, systematic error may be introduced because the constructs that individual informants are reporting on have different psychometric properties. Estimation of reliability is frequently indexed by Cronbach’s alpha (Cronbach, 1951). However, alpha is more correctly interpreted as a measure of internal consistency (Sijtsma, 2009). It does not provide information about the specific measurement structure of the items comprising a test/scale.

To evaluate this possibility, more sophisticated analytic tools are necessary. For example, confirmatory factor analysis (CFA) can evaluate measurement properties such as how items relate to constructs. Extensions of CFA have been developed to test whether measurement properties of constructs are consistent across informants (Olino and Klein, 2015) and assessment waves (Widaman et al., 2010). These methods have been termed measurement invariance (MI; Meredith, 1993).

There are multiple levels of MI that reflect increasingly strict model properties, and address different psychometric questions (Widaman et al., 2010; Millsap, 2011). A fundamental requirement is that the same items are associated with the same construct across units (e.g., informants and time). Simply stated, do the same items load on the same factors when assessed in the different units. This is referred to as configural invariance. If the items assessing what are purportedly the same constructs differ across groups, the items have different meanings within each group. Next, it is important that the magnitude of the associations between the items and the underlying construct is the same across groups (i.e., are the factor loadings for each factor comparable when assessed within the different groups?). This is referred to as metric invariance. Finally, the probability of item endorsement should be the same across groups (Reise et al., 1993; Vandenberg and Lance, 2000). This is referred to as scalar invariance. When configural, metric, and scalar invariance are established for a particular measure across groups, scale scores can be considered to reflect the same psychometric quantities among the groups. Thus, it is critical to evaluate whether lack of MI is contributing to reduced associations between parents and children. However, complete MI imposes highly rigorous assumptions (i.e., equality of all factor loadings and item thresholds across informants). Consequently, there has been increasing attention to the presence of partial MI that specifies invariance on parameters for some, but not all, items (Byrne et al., 1989). This approach has gained prominence and has permitted meaningful comparisons when full MI fails (Steinmetz, 2013).

In the present study, we examine MI across maternal- and child-reports of youth anxiety symptoms when children are ages 9 and 12. Thus, we are able to describe differences in MI across this 3-year developmental span. We also present analyses examining MI across time for maternal- and child-reports separately.

In light of the consistently modest agreement between maternal and child reports of symptomatology, we expect to find a lack of MI across informants at both assessment waves. We do not posit whether this is due to differences in factor loadings or thresholds. However, we expect there to be stronger support for MI across time within informants as there is evidence for longitudinal stability of youth anxiety (Prenoveau et al., 2011). In instances when full MI fails, we examine partial MI that permits some flexibility in the models.

## Materials and Methods

### Participants and Procedure

Participants were from a larger sample of 559 children and their families living in a suburban community who were participating in the Stony Brook Temperament Study, a longitudinal study of temperament and psychopathology, which began when children were 3 years old (Olino et al., 2010a). Potential participants were identified using a commercial mailing list and screened by telephone. Families with a 3-year-old child who lived with an English-speaking biological parent within 20 contiguous miles of Stony Brook, New York and did not have significant medical conditions or developmental disabilities were included. Of the 815 identified eligible families, 68.5% entered the study. No significant differences were found between families who did and did not participate on child sex and race/ethnicity, and parental marital status and education. Informed and written consent was obtained from the parent prior to participation. The study was approved by the institutional review board at Stony Brook University, and families were compensated for their participation. At the second wave of the study, 3 years later, 50 additional minority families were recruited to increase racial/ethnic diversity (total *N* = 609; Bufferd et al., 2012).

At the age 9 visit, 487 mothers (80.0%) and 481 youth (79.0%) completed the measures of youth anxiety symptoms used in this study; a mother or child from 492 families (80.8%) participated. At the age 12 visit, 468 mothers (76.8%) and 470 youth (77.2%) completed these measures; a mother or child from 479 families (78.7%) participated. The mean age of the children was 9.18 years (*SD* = 0.40) at the 9-year assessment and 12.66 (*SD* = 0.46) at the 12-year assessment. Approximately half the children were female (9-year visit: 226, 45.9%; 12-year visit: 225, 47.0%) and the majority were White/non-Hispanic (9-year visit: 390, 79.3%; 12-year visit: 381, 79.5%). At the time of the 12-year visit, most mothers were married (373, 77.9%) and approximately half had graduated from college (279; 58.2%), and the median income bracket was $100,000–$119,999. Youth who participated at age 9 did not differ from those participating at age 3 on child sex, race, or total or externalizing behavior problems, as assessed by maternal reports on the Child Behavior Checklist (Achenbach and Rescorla, 2001; all *p*s > 0.05). However, youth who did not continue with the study at age 9 had higher levels of internalizing problems at age 3 than those who continued with the study, though the effect is small [*t*(547) = 4.69, *P* < 0.05, *d* = 0.09].

### Measures

Children and their parents completed the 41-item youth self-report and parent-report versions, respectively, of the Screen for Childhood Anxiety Related Disorders (SCARED; Birmaher et al., 1997, 1999). Children and their parents are asked to rate the presence of anxiety symptoms in the child over the past 3 months on a three-point scale (0 = not true or hardly ever true; 1 = somewhat true or sometimes true; 2 = very true or often true). The SCARED is made up of five factor-analytically derived subscales: panic/somatic, general anxiety, separation anxiety, social phobia, and school phobia. These subscales reflect anxiety disorder symptoms as conceptualized in the DSM-IV-TR. Each factor has been shown to have good internal consistency and test–retest reliability (range of α: 0.78–0.87; Birmaher et al., 1999; intraclass correlation across time for each scale ranged from 0.70−0.80; Birmaher et al., 1997).

### Statistical Analyses

**FIGURE 1:**
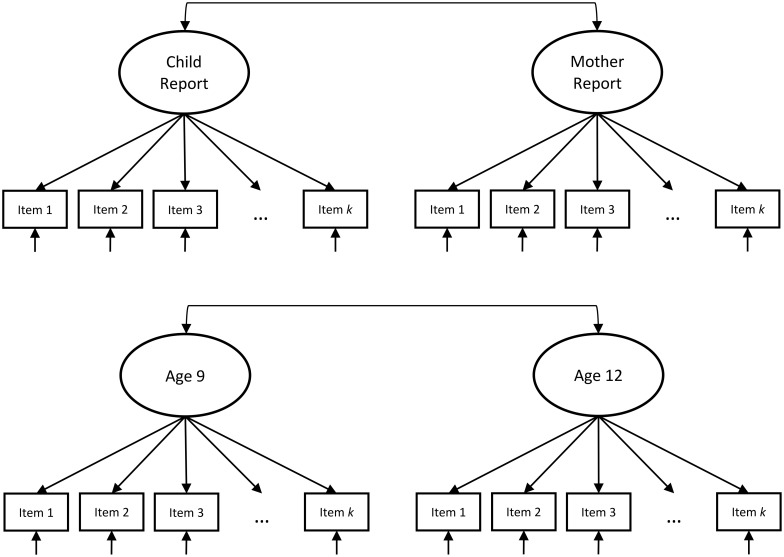
Schematic configural invariance models tested for inter-informant models (top) and across time (bottom).

In line with a model building approach and to identify whether one-factor models were appropriate for testing, we estimated a series of initial single-factor CFAs separately for youth self- and parent-reports at the ages 9 and 12 waves. Items from the panic/somatic, general anxiety, and social phobia subscales were included in models reflecting each of these constructs, respectively. Next, models were fit sequentially to evaluate MI and we continued testing for MI only when there was evidence that a one-factor model for each was an acceptable fit to the data. We followed the same logical progression of testing MI across informants as is used in examinations of longitudinal invariance (Widaman et al., 2010) with minor modifications. We tested first for configural invariance (schematic models for configural invariance models are displayed in **Figure 1**), or whether the pattern of significant (i.e., non-zero) factor loadings is similar across youth and parent-reports. We estimated models for each of the subscales including a single factor for youth and a single factor maternal-reports simultaneously while permitting the factors to be correlated. These models were specified freely estimating all factor loadings and fixing the latent variable variance at 1 for purposes of model identification. Next, we tested for metric invariance, or whether factor loadings for each item are equal across informants. In these models, we freely estimated the variance of the maternal-report latent factor as fixing factor loadings to be equal across informants permits this constraint to be relaxed for one informant. Finally, we tested for scalar invariance, or whether the probability of item endorsement is similar across informants, by constraining the thresholds across informants to be equal. In these models, we freely estimated the mean of the maternal-report latent factor as fixing thresholds to be equal across informants permits this constraint to be relaxed for one informant. If all three types of invariance hold, this indicates that the scales measure the same constructs across reporters on the same scale. Thus, differences in mean trait levels can be interpreted as true score differences, as opposed to differences in measurement.

For models that did not achieve full MI, we tested partial MI, which identifies whether some, but not all, items are invariant across informants and/or time. We examined the presence of comparable factor loadings using the MODEL CONSTRAINT command in Mplus to assess differences in configural invariance. When factor loadings were identified that did not significantly differ at *P* < 0.05, a partial metric invariant model was estimated that included equality constraints on those factor loadings. In this partial metric invariance model, we used the MODEL CONSTRAINT command that tests whether the difference between specified parameters significantly differ, to examine the presence of comparable item thresholds. When item thresholds were identified that did not significantly differ at *P* < 0.05, a partial scalar invariant model was estimated that included equality constraints on those item thresholds.

All models were estimated in Mplus version 8 (Muthén and Muthén, 1998–2017) using the weighted least squares estimator (WLSMV; Flora and Curran, 2004), which is a robust estimator suited for modeling binary data. There were low rates of responses in the highest response category (i.e., “very true or often true”) on many items. Specifically, for 34 (82.9%) items at both ages 9 and 12, 5% or fewer of parents endorsed the highest category. Similarly, for 7 (17.1%) items at age 9, and 23 items (56.1%) at age 12, 5% or fewer of children endorsed the most severe response option. Consequently, the top two item response categories were collapsed, making all items binary. We evaluated models on two goodness of fit indices. Specifically, we used the comparative fit index (CFI; Bentler, 1990) and Root Mean Square Error of Approximation (RMSEA; Steiger, 1990). Although cut-offs are somewhat arbitrary (Marsh et al., 2004), current conventions suggest that excellent model fit is indicated by CFI values ≥ 0.95 (Hu and Bentler, 1999) and RMSEA values ≤0.05 (MacCallum et al., 2006); good fit is indicated by CFI greater than 0.90 and a RMSEA between 0.05 and 0.10.

We estimated configural (similar pattern of factor loadings across groups), metric (equality of factor loadings across groups), and scalar (equality of thresholds across groups) for comparisons between maternal- and child-reports. In addition to testing MI across informants, we also tested the same sequence of models for evaluating longitudinal MI in each informant, separately. Model fit comparisons were evaluated by investigating change in both CFI and RMSEA using Chen’s (2007) guidelines. Chen (2007) recommended interpreting reductions in CFI of 0.01 and RMSEA of 0.015 as indicating non-invariance (i.e., failure to demonstrate MI). When the RMSEA and CFI changes led to different conclusions, we relied on the more conservative index to inform interpretations.

## Results

### Measurement Models for Informant and Age

Initial models estimated one-factor models for each of the SCARED subscales for child self- and maternal-reports at ages 9 and 12. These models were estimated to identify scales that fit the data well enough to pursue tests of MI. **Table 1** displays overall fit for each of the models tested. For age 9 data, one-factor models demonstrated excellent fit for child-reported generalized anxiety disorder (GAD), panic, and social phobia and demonstrated a good fit for maternal-reported GAD, panic, and separation anxiety. For age 12 data, one-factor models demonstrated excellent fit for child-reported panic and good fit for GAD and social phobia, and demonstrated excellent fit for maternal-reported panic and good fit for GAD, separation anxiety, and social phobia. One-factor models for child-reported separation anxiety were poor fits to the data at each time point. Model fit for school avoidance was also less than adequate. For child reports at age 12 and mother reports at age 9, the CFI was acceptable, but the RMSEA was greater than 0.10. In addition, the model for maternal-report of school avoidance at age 12 failed to provide an admissible solution. Owing to the brevity of the school phobia scale, the school avoidance models included only four observed indicators, which may have led to model instability.

**Table 1:** Initial model fit for child self- and maternal-report of SCARED subscales at ages 9 and 12.

	Age 9						Age 12				
	
	Child self-report										
	χ^2^	*df*	*P*	CFI	RMSEA	χ^2^	*df*	*P*	CFI	RMSEA
GAD	37.93	27	0.08	0.987	0.029 (0−0.049)	98.09	27	0.00	0.941	0.075 (0.059−0.091)
Panic	64.05	65	0.51	1.000	0.000 (0.000−0.026)	79.32	65	0.11	0.982	0.022 (0.000−0.037)
School	11.70	2	0.00	0.941	0.100 (0.05−0.159)	35.51	2	0.00	0.914	0.189 (0.137−0.246)
Separation anxiety	134.76	20	0.00	0.868	0.109 (0.092−0.127)	121.30	20	0.00	0.827	0.104 (0.086−0.122)
Social anxiety	28.31	14	0.01	0.978	0.046 (0.021−0.071)	51.44	14	0.00	0.961	0.075 (0.054−0.098)

**Maternal-report**										

GAD	138.02	27	0.00	0.943	0.092 (0.077−0.107)	73.55	27	0.00	0.974	0.061 (0.044−0.078)
Panic	161.52	65	0.00	0.916	0.055 (0.045−0.066)	93.32	65	0.01	0.961	0.031 (0.015−0.044)
School	23.69	2	0.00	0.944	0.149 (0.099−0.206)	NA	NA	NA	NA	NA
Separation anxiety	109.92	20	0.00	0.910	0.096 (0.079−0.114)	58.93	20	0.00	0.911	0.064 (0.046−0.084)
Social anxiety	84.18	14	0.00	0.985	0.101 (0.081−0.123)	43.47	14	0.00	0.987	0.067 (0.045−0.09)

*GAD, generalized anxiety disorder symptoms; panic, panic disorder symptoms; school, school phobia symptoms; separation anxiety, separation anxiety disorder symptoms; and social anxiety, social anxiety symptoms.*

As child-report separation anxiety provided poor fit to the data at ages 9 and 12, we did not assess MI for the youth reports on this subscale. However, as maternal reports of separation anxiety demonstrated good fit, we examined longitudinal invariance for mothers’ reports on this subscale. Due to the problematic fit of the school avoidance models, we did not conduct any MI analyses on this subscale. All model parameters are available in the Supplementary Materials.

### Tests of MI: Child- and Maternal-Reports at Age 9

The configural invariance model for GAD across youth self- and maternal-reports was a good fit to the data (**Table 2**). Likewise, the fit of the metric invariance model was good, and imposing constraints on the factor loadings did not markedly diminish model fit. However, when imposing constraints on the item thresholds across informants, model fit diminished substantially. Comparisons identified three item thresholds that did not significantly differ across informants. Estimating a partial scalar invariant model that constrained those three item thresholds to equality yielded good model fit. Thus, this model supports partial scalar MI.

**Table 2:** Tests of MI between child self- and maternal-reports at age 9.

	χ^2^	*df*	*P*	CFI	RMSEA	ΔCFI	ΔRMSEA
**GAD**		
Configural	252.433	134	<0.001	0.951	0.042 (0.034−0.050)		
Full metric	287.766	142	<0.001	0.940	0.046 (0.038−0.053)	−0.011	0.004
Full scalar	411.928	150	<0.001	0.892	0.060 (0.053−0.066)	−0.048	0.014
Partial scalar^a^	292.481	144	<0.001	0.939	0.046 (0.038−0.053)	−0.001	0.000
**Panic**		
Configural	526.00	298	<0.001	0.864	0.039 (0.034−0.045)		
**Social**		
Configural	157.74	76	<0.001	0.984	0.047 (0.036−0.057)		
Full metric	168.81	82	<0.001	0.983	0.046 (0.036−0.056)	−0.001	−0.001
Full scalar	191.03	88	<0.001	0.980	0.049 (0.039−0.058)	−0.003	0.003

*GAD, generalized anxiety disorder symptoms; panic, panic disorder symptoms; and social anxiety, social anxiety symptoms. Changes in CFI and RMSEA are calculated as differences between the metric invariance model relative to the configural invariance model and between the scalar invariance model relative to the metric invariance model. ^a^In this model, three of nine threshold parameters were constrained to be equal. This model is compared with the full-metric model.*

The configural invariance models for panic disorder across youth self- and maternal-reports were a poor fit to the data. Thus, further tests of metric and scalar invariance were not pursued.

The fit for the configural invariance model for social phobia across youth self- and maternal-reports was good. Likewise, the metric invariance model was a good fit to the data, and imposing constraints on the factor loadings did not markedly diminish model fit. Similarly, imposing constraints on the item thresholds across informants did not substantially diminish model fit, supporting full-scalar MI.

### Tests of MI: Child- and Maternal-Reports at Age 12

The configural invariance model for GAD across youth self- and maternal-reports at age 12 was a good fit to the data (**Table 3**). Likewise, the fit of the metric invariance model was good, and imposing constraints on the factor loadings did not markedly diminish model fit. However, when imposing constraints on the item thresholds across informants, model fit diminished substantially, failing to support scalar invariance. Comparisons identified only one item threshold that did not significantly differ across informants. Thus, this model also failed to support partial scalar MI.

**Table 3:** Tests of MI between child self- and maternal-reports at age 12.

	χ^2^	*df*	*P*	CFI	RMSEA	ΔCFI	ΔRMSEA
**GAD**		
Configural	285.64	134	<0.001	0.944	0.049 (0.041−0.056)		
Full metric	315.25	142	<0.001	0.936	0.050 (0.043−0.058)	−0.008	0.001
Full scalar	447.81	150	<0.001	0.89	0.064 (0.058−0.071)	−0.046	0.014
Partial scalar^a^	315.25	142	<0.001	0.936	0.050 (0.043−0.058)	0.046	−0.014
**Panic**		
Configural	395.49	298	<0.001	0.91	0.026 (0.019−0.033)		
Full metric	414.35	310	<0.001	0.903	0.027 (0.019−0.033)	−0.007	0.001
Full scalar	907.12	335	<0.001	0.47	0.060 (0.055−0.064)	−0.433	0.033
**Social**		
Configural	257.64	76	<0.001	0.941	0.071 (0.061−0.080)		
Full metric	253.94	79	<0.001	0.943	0.068 (0.059−0.077)	0.002	−0.003
Full scalar	297.77	88	<0.001	0.932	0.071 (0.062−0.079)	−0.011	0.003

*GAD, generalized anxiety disorder symptoms; panic, panic disorder symptoms; and social anxiety, social anxiety symptoms. Changes in CFI and RMSEA are calculated as differences between the metric invariance model relative to the configural invariance model and between the scalar invariance model relative to the metric invariance model. The model with best statistical fit is highlighted in bold. ^a^In this model, one of nine threshold parameters were constrained to be equal. This model is compared to the full-metric model.*

The configural invariance model for panic disorder demonstrated adequate fit. Including constraints on factor loadings across informants to test metric invariance yielded a model with an adequate fit to the data and did not markedly differ from the configural invariance model. However, when including constraints on item thresholds to test for scalar invariance, model fit was poor and was reduced relative to the metric invariance model. Moreover, all item thresholds significantly differed across informants, hence there was no basis for evaluating partial scalar invariance.

The configural invariance model for social phobia across youth self- and maternal-reports was a good fit to the data. Likewise, the fit of the metric invariance model was good, and imposing constraints on the factor loadings did not markedly diminish model fit. Finally, after imposing constraints on the item thresholds across informants, model fit was not substantially diminished. Thus, this model supports full-scalar MI.

### Tests of MI: Child-Reports Across Ages 9 and 12

The fit for the configural invariance model for GAD for youth self-reports across ages 9 and 12 was excellent (**Table 4**). Likewise, the metric invariance model was an excellent fit to the data as imposing constraints on the factor loadings did not markedly diminish model fit. When imposing constraints on the item thresholds across informants to test scalar invariance, overall model fit was still good; however, model fit was diminished relative to the metric invariance model. Comparisons identified only two item thresholds that did not significantly differ across informants. This partial scalar invariance model yielded excellent model fit. However, with only two invariance item intercepts, this model failed to sufficiently support partial scalar MI.

**Table 4:** Tests of MI for child self-reports across ages 9 and 12.

	χ^2^	*df*	*P*	CFI	RMSEA	ΔCFI	ΔRMSEA
**GAD**							
Configural	201.47	134	<0.001	0.963	0.031 (0.022−0.04)		
Full metric	219.00	142	<0.001	0.958	0.033 (0.024−0.041)	−0.005	0.002
Full scalar	273.10	150	<0.001	0.933	0.040 (0.032−0.048)	−0.025	0.007
Partial scalar^a^	219.41	143	<0.001	0.958	0.032 (0.024−0.041)	0.025	−0.008
**Panic**		
Configural	324.73	298	<0.001	0.983	0.013 (0.000−0.022)		
Full metric	364.34	310	<0.001	0.964	0.019 (0.008−0.026)	−0.019	0.006
Partial metric^b^	328.83	307	<0.001	0.986	0.012 (0.000−0.021)	0.022	−0.007
Scalar^c^	442.99	316	<0.001	0.917	0.028 (0.022−0.034)	−0.069	0.016
Partial scalar^d^	334.14	310	<0.001	0.984	0.012 (0.000−0.021)	−0.002	0.000
**Social**		
Configural	143.92	76	<0.001	0.955	0.042 (0.031−0.052)		
Full metric	189.32	82	<0.001	0.929	0.051 (0.041−0.060)	−0.026	0.009
Partial metric^e^	142.38	78	<0.001	0.957	0.040 (0.030−0.051)	0.002	−0.002
Scalar^f^	150.62	80	<0.001	0.953	0.042 (0.031−0.052)	−0.004	0.002

*GAD, generalized anxiety disorder symptoms; panic, panic disorder symptoms; and social anxiety, social anxiety symptoms. Changes in CFI and RMSEA are calculated as differences between the metric invariance model relative to the configural invariance model and between the scalar invariance model relative to the metric invariance model. The model with best statistical fit is highlighted in bold. ^a^In this model, two of nine threshold parameters were constrained to be equal. This model is compared to the full-metric model. ^b^In this model, 10 of 13 factor loading parameters were constrained to be equal. This model is compared to the configural invariance model. ^c^In this model, 3 of 13 threshold parameters were freely estimated across time. This model is compared to the partial metric model. ^d^In this model, 4 of 13 threshold parameters were constrained to be equal. This model is compared to the partial metric model. ^e^In this model, three of seven factor loading parameters were constrained to be equal. This model is compared to the configural invariance model. ^f^In this model, three of seven threshold parameters are constrained across time. This model is compared to the partial metric model.*

The fit for the configural invariance model for panic disorder for youth self-reports across ages 9 and 12 was excellent (**Table 4**). The metric invariance model was also an excellent fit to the data. However, there was a substantial reduction in model fit as indexed by the CFI and a more modest reduction in fit according to the RMSEA. Comparisons identified three factor loadings that differed across age. Model fit for the partial metric invariance model was an excellent fit to the data. As only partial metric invariance was supported, when estimating scalar invariance, thresholds for items that did not evince equal factor loadings across time were freely estimated. After imposing constraints on the other item thresholds across time, overall model fit was still good; however, model fit was diminished relative to the partial metric invariance model. Comparisons identified four item thresholds that did not significantly differ across time. This partial scalar invariance model yielded excellent model fit.

The fit for the configural invariance model for social phobia for youth self-reports across ages 9 and 12 was an excellent fit to the data. The fit of the metric invariance model was also good. However, there was a substantial reduction in model fit as indexed by the CFI, and a modest reduction in the RMSEA. Comparisons identified three factor loadings that did not statistically differ across age. Model fit for the partial metric invariance model was an excellent fit to the data. As only partial metric invariance was supported, when estimating scalar invariance, item thresholds for items that did not evince equal factor loadings across time were freely estimated. Three item thresholds were constrained across time. After imposing constraints on the item thresholds across informants to test for scalar invariance, model fit was not substantially diminished, supporting partial scalar MI.

### Tests of MI: Maternal-Reports Across Ages 9 and 12

The configural invariance model for GAD for mother-reports across ages 9 and 12 was an excellent fit to the data (**Table 5**). The fit of the metric invariance model was good, and imposing constraints on the factor loadings did not markedly diminish model fit, supporting metric invariance. After imposing constraints on the item thresholds across informants, overall model fit was still good and showed a minor reduction in model fit as indexed by the CFI and a trivial reduction in the RMSEA. Thus, scalar MI was supported.

**Table 5:** Tests of MI for maternal-reports across ages 9 and 12.

	χ^2^	*df*	*P*	CFI	RMSEA	ΔCFI	ΔRMSEA
**GAD**		
Configural	319.88	134	<0.001	0.950	0.052 (0.045−0.059)		
Full metric	333.82	142	<0.001	0.948	0.051 (0.044−0.058)	−0.002	−0.001
Full scalar	396.99	150	<0.001	0.933	0.057 (0.050−0.063)	−0.015	0.006
**Panic**		
Configural	501.40	298	<0.001	0.899	0.036 (0.031−0.042)		
**Separation**		
Configural	256.54	103	<0.001	0.900	0.054 (0.046−0.062)		
Full metric	270.92	110	<0.001	0.896	0.053 (0.045−0.061)	−0.004	−0.001
Partial metric^a^	252.09	109	<0.001	0.907	0.050 (0.042−0.059)	0.007	−0.004
Scalar^b^	307.39	117	<0.001	0.876	0.056 (0.049−0.064)	−0.031	0.006
**Social**		
Configural	234.44	76	<0.001	0.978	0.064 (0.054−0.073)		
Full metric	348.49	82	<0.001	0.963	0.079 (0.071−0.088)	−0.015	0.015
Partial metric^c^	211.41	81	<0.001	0.982	0.056 (0.047−0.065)	0.004	−0.008
Scalar^d^	219.18	85	<0.002	0.981	0.055 (0.046−0.064)	−0.001	−0.001

*GAD, generalized anxiety disorder symptoms; panic, panic disorder symptoms; separation anxiety, separation anxiety disorder symptoms; social anxiety, social anxiety symptoms. Changes in CFI and RMSEA are calculated as differences between the metric invariance model relative to the configural invariance model and between the configural invariance model relative to the metric invariance model. The model with best statistical fit is highlighted in bold. ^a^In this model, seven of eight factor loading parameters were constrained to be equal. This model is compared to the configural invariance model. ^b^In this model, 3 of 13 threshold parameters were freely estimated across time. This model is compared to the partial metric model. ^c^In this model, six of seven factor loading parameters were constrained to be equal. This model is compared to the configural invariance model. ^b^In this model, one of seven threshold parameters were freely estimated across time. This model is compared to the partial metric model.*

The configural invariance model for panic disorder was an adequate fit to the data. However, there were problems in estimating the metric and scalar invariance models due to low endorsement rates of item response options across multiple items (i.e., empty cells in bivariate distributions). Thus, those models could not be adequately tested.

The configural invariance model for separation anxiety was good. The metric invariance model marginally reduced model fit, but it was enough to result in a less than adequate fit to the data. Comparisons of factor loadings identified one parameter that statistically differed across time. Model fit for the partial metric invariance model was good, supporting partial metric invariance. After adding constraints on item thresholds across time, model fit was reduced and demonstrated a poor fit to the data. Comparisons of item thresholds revealed that all parameters differed across time. Thus, there was no support for partial scalar invariance.

The fit for the configural invariance model for social phobia for maternal-reports across ages 9 and 12 was excellent. The metric invariance model was also an excellent fit to the data. However, there was a reduction in model fit as indexed by the CFI and the RMSEA. Comparisons of factor loadings identified six (of seven) factor loadings that did not statistically differ across age. Fit for the partial metric invariance model was excellent, supporting partial metric invariance. As only partial metric invariance was supported, when estimating scalar invariance, the item threshold for the item that did not evince equal factor loadings across time was freely estimated. After imposing constraints on the item thresholds across time to test for scalar invariance, overall model fit was excellent and the model did not demonstrate a substantial reduction in fit relative to the partial metric invariant model, supporting scalar invariance.

## Discussion

There has been much previous work examining factors and contexts that influence correspondence between parents’ and their children’s reports of psychopathology (Achenbach et al., 1987; De Los Reyes et al., 2015). However, there has been much less research examining measurement properties between informants that could influence the comparability of reports of youth behavior. Similarly, there has been little attention to examining MI across time, which is critical to understanding whether mean-level changes across time are contaminated by changes in measurement properties of items (Widaman et al., 2010). In the present study, we used the subscales from the SCARED to examine overall fit of each anxiety construct in each informant and at each assessment. Then we examined MI between mothers and their children at ages 9 and 12. Finally, we examined invariance for each rater from middle childhood to early adolescence. Overall, full MI was supported between children and their mothers for social anxiety at both ages 9 and 12, but not for any other SCARED subscale. We found support for partial metric invariance across mothers and children at age 9 for GAD. Longitudinally, full-scalar invariance was found for maternal reports of GAD over time and partial scalar invariance was supported for child reported panic and social anxiety and for maternal reported separation anxiety across the two waves.

Thus, we found support for full-scalar invariance across informants for only one SCARED subscale-social anxiety. This indicates that direct comparisons of mean levels of child and maternal reported anxiety symptoms are valid only for this scale of the SCARED.

To demonstrate “strong enough” measurement properties, there has to be consistent evidence supporting at least partial metric invariance across informants at both ages 9 and 12 (Marsh and Grayson, 1994). This indicates that a subset of items reflect the same target latent construct across mothers and their children. Thus, the construct reported on by each informant is conceptually similar in form and reflects rank-order associations among like-constructs. This suggests that for the scales demonstrating at least partial metric invariance inter-informant associations are meaningful. This condition was satisfied by the GAD scale at both ages 9 and 12. However, the lack of scalar invariance precludes comparing mean levels of generalized anxiety across informants (Millsap, 2011).

Panic, school avoidance, and separation anxiety showed the least evidence for MI. Although the panic symptom models demonstrated good fit to the data in our four preliminary models (i.e., separate informant and assessment; **Table 1**), tests of configural invariance across informant yielded poor fit to the data at age 9 and marginal fit to the data at age 12. Moreover, the fit of configural invariance models for school avoidance and separation anxiety was poor. Fit of these models may have been impacted by the developmental level of the children in the study. School avoidance and separation anxiety are typically observed at higher levels earlier in development. Thus, the coherence of the items in later childhood may be poorer than earlier in development (Hayward et al., 2000; Mathyssek et al., 2012). Moreover, incidence of panic continues to rise through adolescence (Beesdo et al., 2009) and item functioning may continue to change.

Examining the pattern of differences in factor loadings and thresholds between child and maternal reports, there is a consistent pattern of maternal reports having larger factor loadings and thresholds. Stronger factor loadings for maternal scores suggest that their ratings have greater precision and are better at discriminating between children with high and low levels of anxiety. Higher item thresholds for maternal than child-reported items suggest that symptoms need to be more severe for mothers to rate them as present relative to children. Taken together, these findings pose significant challenges to comparing levels of anxiety across mothers and youth. With only a few exceptions, these results argue against direct comparisons of mothers’ and youth’s anxiety ratings.

Our models testing longitudinal invariance demonstrated greater, albeit modest, support for MI over time for each informant taken separately. Maternal reports of youth GAD achieved full-scalar invariance, suggesting that scores from this scale are comparable from middle childhood to early adolescence. Child-reports of panic and social anxiety and maternal-reports of separation anxiety demonstrated a good fit to the data and partial scalar invariance. For these scales, there were some items that demonstrate invariance across time, permitting longitudinal comparisons of latent mean-level differences on the full set of items or examining mean-level differences on the subset of items. These comparisons should reflect true changes in the constructs, rather than being conflated with changes in item properties. Child-report of GAD and maternal-report of social anxiety each had a small number of items with invariant factor loadings and threshold. Based on these results, there should be concern about relying on this set of items/scales to assess developmental changes on dimensions of anxiety symptoms, particularly when relying on child self-reports, and provide little basis for combining these ratings. However, our findings raise the question of whether these subscales evidence MI invariance over shorter periods of time and from pre- to post-test in evaluations of interventions. If psychometric functioning is changing over time, it may not be possible to distinguish intervention effects from measurement changes.

In our work, we focused on the primary, lower-order scales that demonstrated at least adequate fit for a one-factor model. In this evaluation, school phobia and some of the assessments of separation anxiety were not unitary factors. Thus, we did not evaluate these dimensions for MI. This suggests that more in-depth analysis of these dimensions is warranted, although there are only four items on the school phobia subscale, restricting alternative modeling strategies to yield better fit. Alternatively, because school phobia and separation anxiety are most common in early childhood, there may have been limited variability in responses for these dimensions at ages 9 and 12. Earlier assessments of school phobia and separation anxiety may have greater variability (Merikangas et al., 2010) and could lead to better fitting models. Examination of other instruments (e.g., the RCADS; Chorpita et al., 2000) across informants and time would provide leverage to determine whether this is a measure-specific or construct assessment challenge.

The present study employed an underutilized lens to better understand sources of discrepancy between child- and parent-reports of anxiety, as well as instability of anxiety symptoms from middle childhood to early adolescence. We employed a relatively large sample of mothers and youth who reported on multiple dimensions of anxiety symptomatology in middle childhood and early adolescence. However, our work has some limitations. First, our data came from a community sample with modest levels of symptomatology. Further, we had truncated ranges of item endorsement and collapsed our highest endorsement categories. We are unsure how this may have affected the findings. Second, we used only a single measure of anxiety, albeit one of the most frequently employed with children and adolescents. It is possible that other measures may demonstrate different levels of robustness across informants or longitudinal assessments. Third, we relied solely on comparisons between mothers and children. It is important to consider whether other caregivers (e.g., fathers) and teachers report on the same constructs of behavior problems in children. Fourth, we focused on individual subscales, rather than the total SCARED score. Thus, our work emphasizes these anxiety domains, but does not speak to the similarity in the overall structure of anxiety between informants and across time. Additional analyses would be necessary that focus on the broader dimensional model of the SCARED as a whole. Here, preliminary multidimensional models for the total SCARED produced good fit at age 9, but only a marginal fit at age 12. Thus, there is some evidence that the general structure may differ across time. Adequate testing of this more complex model would require a larger sample with greater variability in anxiety severity. Fifth, there was some selection for continuing the study when youth had lower levels of internalizing problems at age 3. Though this difference was small.

In sum, our findings illustrate that it is critical to evaluate measurement properties of anxiety symptom rating scales using sophisticated measurement strategies. We found that associations across informants may be compromised by differences in the functioning of items on the scale being examined. In such cases, testing for differences between informants and combining ratings across informants to yield single indices of severity are both inappropriate. However, there was also evidence that measurement functioning for some anxiety dimensions remained consistent over time. Thus, a few of the dimensions of the SCARED are valid for assessing longitudinal change. As it may be difficult to know *a priori* which measures are appropriate for assessing change, there is a pressing need for a comprehensive effort to evaluate MI for the full range of scales commonly used to assess developmental trajectories and response to treatment in child and adolescent clinical psychology and psychiatry.

## Ethics Statement

Informed consent was obtained prior to participation in accordance with the Declaration of Helsinki. The study was approved by the institutional review board at Stony Brook University.

## Author Contributions

TO conceptualized the research questions, drafted the manuscript, and conducted analyses. MF provided assistance in conducting analyses and provided critical feedback on the manuscript. LD provided critical feedback on the manuscript. DK provided substantial contribution to the research design and critical feedback on the manuscript.

## Conflict of Interest Statement

The authors declare that the research was conducted in the absence of any commercial or financial relationships that could be construed as a potential conflict of interest.

## References

[B1] Achenbach T. M. McConaughy S. H. Howell C. T. (1987). Child/adolescent behavioral and emotional problems: implications of cross-informant correlations for situational specificity. *Psychol. Bull.* 101 213–232. 10.1037/0033-2909.101.2.213 3562706

[B2] Achenbach T. M. Rescorla L. (2001). *Manual for the ASEBA School-age Forms & Profiles.* Burlington, VT: University of Vermont.

[B3] Bentler P. M. (1990). Comparative fit indexes in structural models. *Psychol. Bull.* 107 238–246. 10.1037/0033-2909.107.2.238 2320703

[B4] Beesdo K. Knappe S. Pine D. S. (2009). Anxiety and anxiety disorders in children and adolescents: developmental issues and implications for DSM-V. *Psychiatr. Clin. North Am.* 32 483–524. 10.1016/j.psc.2009.06.002 19716988 PMC3018839

[B5] Birmaher B. Brent D. A. Chiappetta L. Bridge J. Monga S. Baugher M. (1999). Psychometric properties of the screen for child anxiety related emotional disorders (SCARED): a replication study. *J. Am. Acad. Child Adolesc. Psychiatry* 38 1230–1236. 10.1097/00004583-199910000-00011 10517055

[B6] Birmaher B. Khetarpal S. Brent D. Cully M. Balach L. Kaufman J. (1997). The screen for child anxiety related emotional disorders (SCARED): scale construction and psychometric characteristics. *J. Am. Acad. Child Adolesc. Psychiatry* 36 545–553. 10.1097/00004583-199704000-00018 9100430

[B7] Bufferd S. J. Dougherty L. R. Carlson G. A. Rose S. Klein D. N. (2012). Psychiatric disorders in preschoolers: continuity from ages 3 to 6. *Am. J. Psychiatry* 169 1157–1164. 10.1176/appi.ajp.2012.12020268 23128922 PMC3513401

[B8] Byrne B. M. Shavelson R. J. Muthén B. (1989). Testing for the equivalence of factor covariance and mean structures: the issue of partial measurement invariance. *Psychol. Bull.* 105 456–466. 10.1177/1073191111419091 21862530

[B9] Chen F. F. (2007). Sensitivity of goodness of fit indexes to lack of measurement invariance. *Struct. Equ. Modeling* 14 464–504. 10.1080/10705510701301834

[B10] Chorpita B. F. Yim L. Moffitt C. Umemoto L. A. Francis S. E. (2000). Assessment of symptoms of DSM-IV anxiety and depression in children: a revised child anxiety and depression scale. *Behav. Res. Ther.* 38 835–855. 10.1016/S0005-7967(99)00130-8 10937431

[B11] Cronbach L. J. (1951). Coefficient alpha and the internal structure of tests. *Psychometrika* 16 297–334. 10.1007/BF02310555

[B12] De Los Reyes A. Augenstein T. M. Wang M. Thomas S. A. Drabick D. A. Burgers D. E. (2015). The validity of the multi-informant approach to assessing child and adolescent mental health. *Psychol. Bull.* 141 858–900. 10.1037/a0038498 25915035 PMC4486608

[B13] De Los Reyes A. Thomas S. A. Goodman K. L. Kundey S. M. (2013). Principles underlying the use of multiple informants’ reports. *Annu. Rev. Clin. Psychol.* 9 123–149. 10.1146/annurev-clinpsy-050212-185617 23140332 PMC4103654

[B14] Feng X. Shaw D. S. Silk J. S. (2008). Developmental trajectories of anxiety symptoms among boys across early and middle childhood. *J. Abnorm. Psychol.* 117 32–47. 10.1037/0021-843X.117.1.32 18266484 PMC2711562

[B15] Flora D. B. Curran P. J. (2004). An empirical evaluation of alternative methods of estimation for confirmatory factor analysis with ordinal data. *Psychol. Methods* 9 466–491. 10.1037/1082-989X.9.4.466 15598100 PMC3153362

[B16] Frank S. J. Van Egeren L. A. Fortier J. L. Chase P. (2000). Structural, relative, and absolute agreement between parents’ and adolescent inpatients’ reports of adolescent functional impairment. *J. Abnorm. Child Psychol.* 28 395–402. 10.1023/A:1005125211187 10949963

[B17] Hayden E. P. Durbin C. E. Klein D. N. Olino T. M. (2010). Maternal personality influences the relationship between maternal reports and laboratory measures of child temperament. *J. Pers. Assess.* 92 586–593. 10.1080/00223891.2010.513308 20954060

[B18] Hayward C. Killen J. D. Kraemer H. C. Taylor C. B. (2000). Predictors of panic attacks in adolescents. *J. Am. Acad. Child Adolesc. Psychiatry* 39 207–214. 10.1097/00004583-200002000-00021 10673832

[B19] Hu L. Bentler P. M. (1999). Cutoff criteria for fit indexes in covariance structure analysis: conventional criteria versus new alternatives. *Struct. Equ. Modeling* 6 1–55. 10.1080/10705519909540118

[B20] Kagan J. (1997). Temperament and the reactions to unfamiliarity. *Child Dev.* 68 139–143. 10.2307/1131931 9084130

[B21] Karver M. S. (2006). Determinants of multiple informant agreement on child and adolescent behavior. *J. Abnorm. Child Psychol.* 34 242–253. 10.1007/s10802-005-9015-6 16514552

[B22] Langberg J. M. Epstein J. N. Simon J. O. Loren R. E. Arnold L. E. Hechtman L. (2010). Parent agreement on ratings of children’s attention deficit/hyperactivity disorder and broadband externalizing behaviors. *J. Emot. Behav. Disord.* 18 41–50. 10.1177/1063426608330792 21691457 PMC3117618

[B23] MacCallum R. C. Browne M. W. Sugawara H. M. (2006). Power analysis and determination of sample size for covariance structure modeling. *Psychol. Methods* 1 130–149. 10.1037/1082-989X.1.2.130 16594765

[B24] Marsh H. W. Grayson D. (1994). Longitudinal stability of latent means and individual differences: a unified approach. *Struct. Equ. Modeling* 1 317–359. 10.1080/10705519409539984

[B25] Marsh H. W. Hau K. T. Wen Z. (2004). In search of golden rules: comment on hypothesis-testing approaches to setting cutoff values for fit indexes and dangers in overgeneralizing Hu and Bentler’s (1999) findings. *Struct. Equ. Modeling* 11 320–341. 10.1207/s15328007sem1103_2

[B26] Mathyssek C. M. Olino T. M. Hartman C. A. Ormel J. Verhulst F. C. Van Oort F. V. (2013). Does the Revised Child Anxiety and Depression Scale (RCADS) measure anxiety symptoms consistently across adolescence? The TRAILS study. *Int. J. Methods Psychiatr. Res.* 22 27–35. 10.1002/mpr.1380 23483654 PMC3801212

[B27] Mathyssek C. M. Olino T. M. Verhulst F. C. van Oort F. V. (2012). Childhood internalizing and externalizing problems predict the onset of clinical panic attacks over adolescence: the TRAILS study. *PLoS One* 7:e51564. 10.1371/journal.pone.0051564 23251576 PMC3520948

[B28] Meredith W. (1993). Measurement invariance, factor analysis and factorial invariance. *Psychometrika* 58 525–543. 10.1007/BF02294825

[B29] Merikangas K. R. He J. P. Burstein M. Swanson S. A. Avenevoli S. Cui L. (2010). Lifetime prevalence of mental disorders in US adolescents: results from the National Comorbidity Survey Replication–Adolescent Supplement (NCS-A). *J. Am. Acad. Child Adolesc. Psychiatry* 49 980–989. 10.1016/j.jaac.2010.05.017 20855043 PMC2946114

[B30] Millsap R. E. (2011). *Statistical Approaches to Measurement Invariance.* New York, NY: Taylor and Francis Group.

[B31] Muthén L. K. Muthén B. O. (1998–2017). *Mplus User’s Guide*, 8th Edn Los Angeles, CA: Muthén & Muthén.

[B32] Najman J. M. Williams G. M. Nikles J. Spence S. Bor W. O’Callaghan M. (2001). Bias influencing maternal reports of child behaviour and emotional state. *Soc. Psychiatry Psychiatr. Epidemiol.* 36 186–194. 10.1007/s001270170062 11518032

[B33] Olino T. M. Klein D. N. (2015). Psychometric comparison of self-and informant-reports of personality. *Assessment* 22 655–664. 10.1177/1073191114567942 25612626 PMC5248555

[B34] Olino T. M. Klein D. N. Dyson M. W. Rose S. A. Durbin C. E. (2010a). Temperamental emotionality in preschool-aged children and depressive disorders in parents: associations in a large community sample. *J. Abnorm. Psychol.* 119 468–478. 10.1037/a0020112 20677836 PMC2989334

[B35] Olino T. M. Klein D. N. Lewinsohn P. M. Rohde P. Seeley J. R. (2010b). Latent trajectory classes of depressive and anxiety disorders from adolescence to adulthood: descriptions of classes and associations with risk factors. *Compr. Psychiatry* 51 224–235. 10.1016/j.comppsych.2009.07.002 20399331 PMC2857532

[B36] Olino T. M. Stepp S. D. Keenan K. Loeber R. Hipwell A. (2014). Trajectories of depression and anxiety symptoms in adolescent girls: a comparison of parallel trajectory approaches. *J. Pers. Assess.* 96 316–326. 10.1080/00223891.2013.866570 24369925 PMC4225067

[B37] Prenoveau J. M. Craske M. G. Zinbarg R. E. Mineka S. Rose R. D. Griffith J. W. (2011). Are anxiety and depression just as stable as personality during late adolescence? Results from a three-year longitudinal latent variable study. *J. Abnorm. Psychol.* 120 832–843. 10.1037/a0023939 21604827 PMC3407811

[B38] Reise S. P. Widaman K. F. Pugh R. H. (1993). Confirmatory factor analysis and item response theory: two approaches for exploring measurement invariance. *Psychol. Bull.* 114 552–566. 10.1037/0033-2909.114.3.552 8272470

[B39] Rothen S. Vandeleur C. L. Lustenberger Y. Jeanprêtre N. Ayer E. Gamma F. (2009). Parent–child agreement and prevalence estimates of diagnoses in childhood: direct interview versus family history method. *Int. J. Methods Psychiatr. Res.* 18 96–109. 10.1002/mpr.281 19507167 PMC6878311

[B40] Sijtsma K. (2009). On the use, the misuse, and the very limited usefulness of Cronbach’s alpha. *Psychometrika* 74 107–120. 10.1007/s11336-008-9101-0 20037639 PMC2792363

[B41] Steiger J. H. (1990). Structural model evaluation and modification: an interval estimation approach. *Multivariate Behav. Res.* 25 173–180. 10.1207/s15327906mbr2502_4 26794479

[B42] Steinmetz H. (2013). Analyzing observed composite differences across groups: is partial measurement invariance enough? *Methodology* 9 1–12. 10.1027/1614-2241/a000049

[B43] Treutler C. M. Epkins C. C. (2003). Are discrepancies among child, mother, and father reports on children’s behavior related to parents’ psychological symptoms and aspects of parent–child relationships? *J. Abnorm. Child Psychol.* 31 13–27. 10.1023/A:1021765114434 12597696

[B44] Van Oort F. Greaves-Lord K. Verhulst F. Ormel J. Huizink A. (2009). The developmental course of anxiety symptoms during adolescence: the TRAILS study. *J. Child Psychol. Psychiatry* 50 1209–1217. 10.1111/j.1469-7610.2009.02092.x 19490305

[B45] Vandenberg R. J. Lance C. E. (2000). A review and synthesis of the measurement invariance literature: suggestions, practices, and recommendations for organizational research. *Organ. Res. Methods* 3 4–70. 10.1177/109442810031002

[B46] Widaman K. F. Ferrer E. Conger R. D. (2010). Factorial invariance within longitudinal structural equation models: measuring the same construct across time. *Child Dev. Perspect.* 4 10–18. 10.1111/j.1750-8606.2009.00110.x 20369028 PMC2848495

[B47] Youngstrom E. Loeber R. Stouthamer-Loeber M. (2000). Patterns and correlates of agreement between parent, teacher, and male adolescent ratings of externalizing and internalizing problems. *J. Consult. Clin. Psychol.* 68 1038–1050. 10.1037/0022-006X.68.6.1038 11142538

